# Case report of severe coronary artery disease complicated by malignant arrhythmia due to inherited thrombophilia

**DOI:** 10.3389/fcvm.2025.1517117

**Published:** 2025-04-09

**Authors:** Jinmei Yu, Lin Zhou, Chengying Yang, Xingyuan Kou, Le Li, Xinrong Fan, Xing Liu

**Affiliations:** ^1^ Department of Cardiovascular Medicine, Southwest Medical University, Luzhou, Sichuan, China; ^2^ Department of Imaging, Southwest Medical University, Luzhou, Sichuan, China

**Keywords:** myocardial infarction, arrhythmia, thrombophilia, cardiac function, coronary artery disease, youth

## Abstract

The principal clinical manifestation of thrombophilia is venous thromboembolism, which is also markedly linked to arterial thrombosis, including myocardial infarction. Nevertheless, patients presenting with an early-onset myocardial infarction are seldom screened for thrombophilic genes, resulting in delayed diagnosis and an unfavourable prognosis. This report presents the case of a young man who suffered an acute myocardial infarction as a result of thrombophilia. The patient had a history of deep vein thrombosis and was genetically tested to carry two thrombophilia susceptibility alleles at the PAI-1 (4G/5G) and MTHFR (C > T) loci. This ultimately resulted in severe coronary artery occlusion, myocardial scarring and frequent episodes of ventricular tachycardia, which had a significant impact on the patient's quality of life. The objective of this report was to enhance clinicians' awareness of embolism susceptibility. It is recommended that young and middle-aged patients with severe coronary artery stenosis undergo screening for embolism.

## Introduction

Thrombophilia refers to a pathological state in which thrombosis and thromboembolism are prone to occur due to various genetic or acquired factors (1). Thrombophilia is more common in patients with unguided venous thromboembolism (VTE), especially in those under 40 years old, with the majority being hereditary (2). Acquired thrombophilia mainly occurs in patients with various acquired diseases or acquired risk factors, due to increased levels of procoagulant proteins, decreased levels of anticoagulant proteins, and altered inflammation/autoimmune mechanisms, which increase the tendency for thromboembolism. Hereditary thrombophilia is commonly caused by mutations in genes such as antithrombin (AT), protein C (PC), protein S (PS), coagulation factor V Leiden (FVL), plasminogen activator inhibitor-1 (PAI-1), and methylenetetrahydrofolate reductase (MTHFR), leading to loss of protein anticoagulant function or enhancement of procoagulant function, ultimately resulting in thromboembolism. And thrombosis is more common in the venous system and less common in arteries (especially coronary arteries). The occurrence of acute myocardial infarction (AMI) is significantly correlated with hypercoagulability and impaired fibrinolysis (3). Therefore, certain types of thrombophilia may also manifest as young onset acute coronary syndrome, ischemic stroke, and other arterial thrombotic events.

Plasminogen activator inhibitor-1 (PAI-1) is a member of serine protease inhibitor (SERPIN) family that acts as the primary inhibitor of two main mammalian plasminogen activators, urinary-type (uPA) and tissue-type (tPA). Research has shown that a deficiency of PAI-1 accelerates the rate of fibrinolysis and bleeding, while an increase in PAI-1 levels can easily lead to intravascular thrombosis (4, 5). The deletion/insertion polymorphism (4G/5G) within the PAI-1 locus can affect the transcriptional expression of this gene, and males with 4G allele are prone to acute myocardial infarction and coronary artery thrombosis (6–8).

Methylenetetrahydrofolate reductase (MTHFR) plays a major role in regulating homocysteine (HC) levels and increases the risk of venous thromboembolism and CAD (9, 10). In addition, genotype is associated with the bioavailability of vascular nitric oxide and the production of superoxide by uncoupling endothelial nitric oxide synthase. It is speculated that MTHFR mutations may also be a high-risk factor for myocardial infarction (11).

In summary, hereditary thrombophilia plays a crucial role in myocardial infarction in specific populations. In addition, cases of coronary thrombosis caused by carrying two susceptibility genes, PAI-1 (4G/5G) and MTHFR (C > T), are extremely rare, and there are great difficulties in screening and diagnosis, which may be related to severe complications and poor prognosis after myocardial infarction. However, there is currently no research reporting on the diagnosis and treatment of young myocardial infarction and serious complications caused by genetic thrombophilia.

This case report demonstrates the genetic testing of a young myocardial infarction patient with thrombophilia and provides follow-up information, in order to provide diagnostic and therapeutic evidence for the treatment and screening of thrombophilia.

## Case presentation

A 25-year-old male was admitted to the hospital due to recurrent episodes of tachycardia. The duration of tachycardia is about 1 h, accompanied by a feeling of obstruction in the throat, sweating, and shortness of breath. It is relieved after rest, and there is no chest pain or tightness. The patient has a 10-year history of smoking. He was diagnosed with deep vein thrombosis in his right lower limb 8 years ago. After taking rivaroxaban orally, the thrombosis was controlled and he stopped taking the medication on his own. In addition, the patient's father suffered from a cerebral infarction, which resulted in limb paralysis.

**Figure 1:**
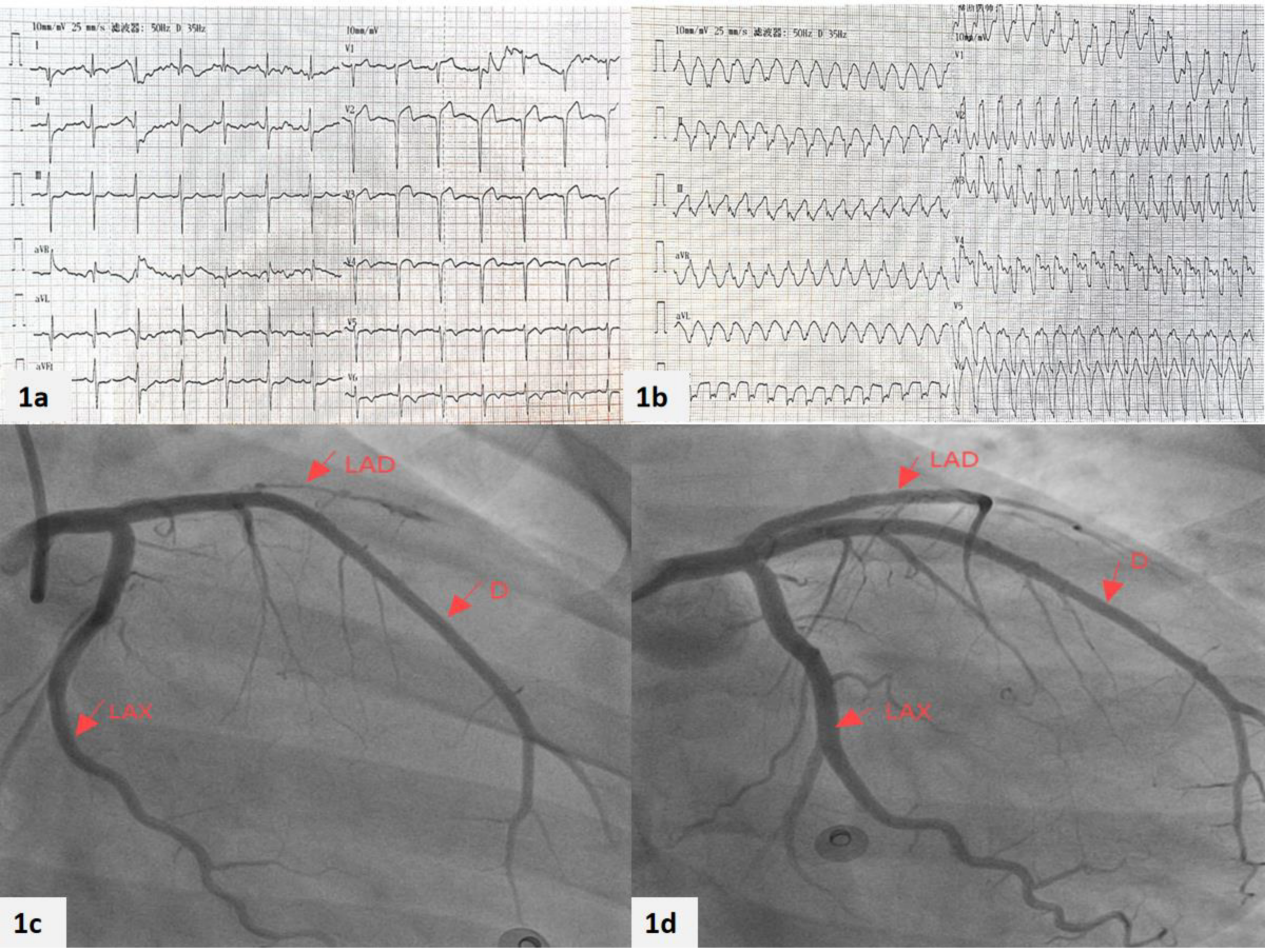
Patient's first admission electrocardiogram combined with coronary angiography and stent placement. The patient's **(a)** electrocardiogram at the time of hospital admission and **(b)** the electrocardiogram during the follow-up examination 2 months later. The results of the patient's coronary angiography **(c)** found that occlusion in the proximal segment of the left anterior descending artery (LAD). Then **(d)** stents were implanted.

**Figure 2:**
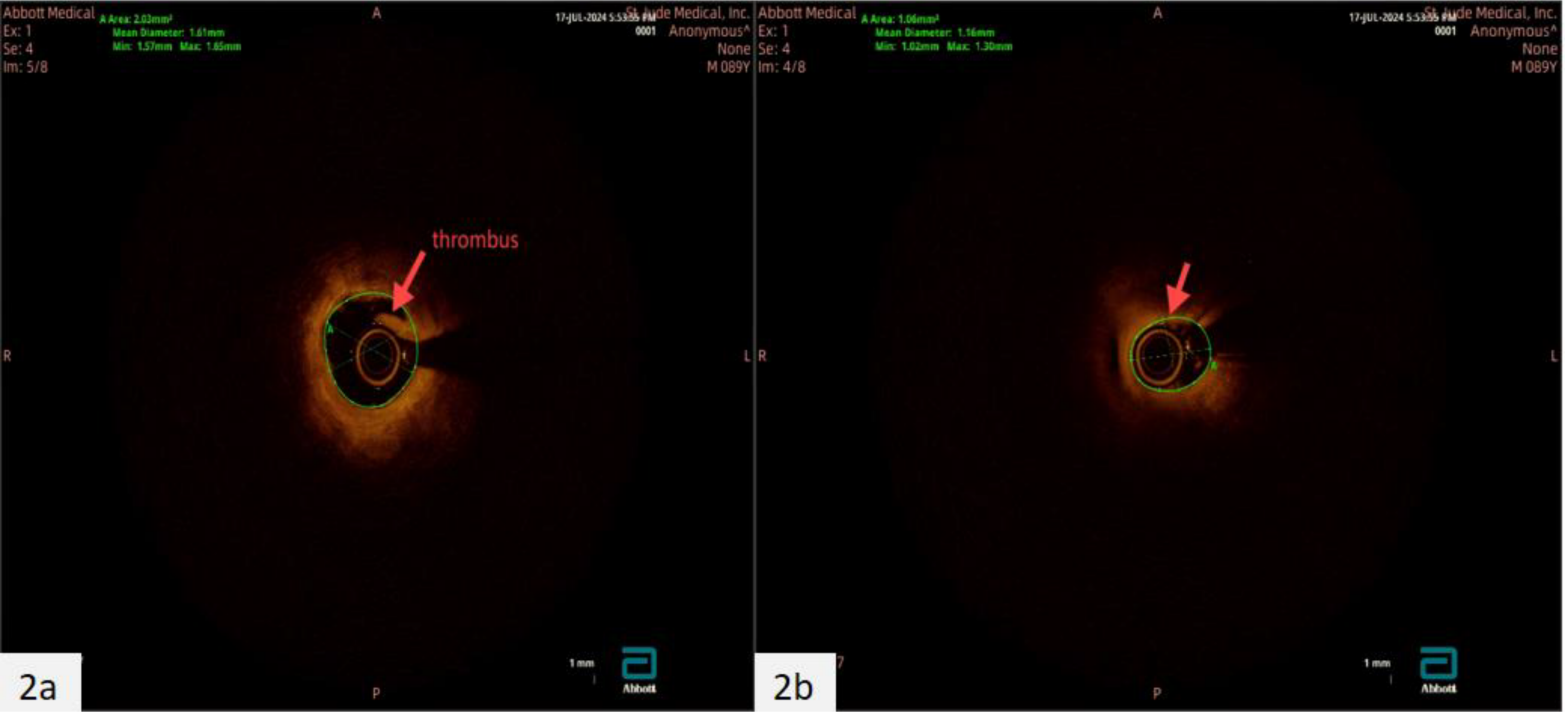
Coronary artery OCT image. There is a large amount of thrombus in the coronary artery of the patient's LAD **(a)**, causing coronary stenosis **(b)** and acute myocardial infarction. The area indicated by the arrow in the figure represents the location of vascular stenosis or thrombus.

**Figure 3:**
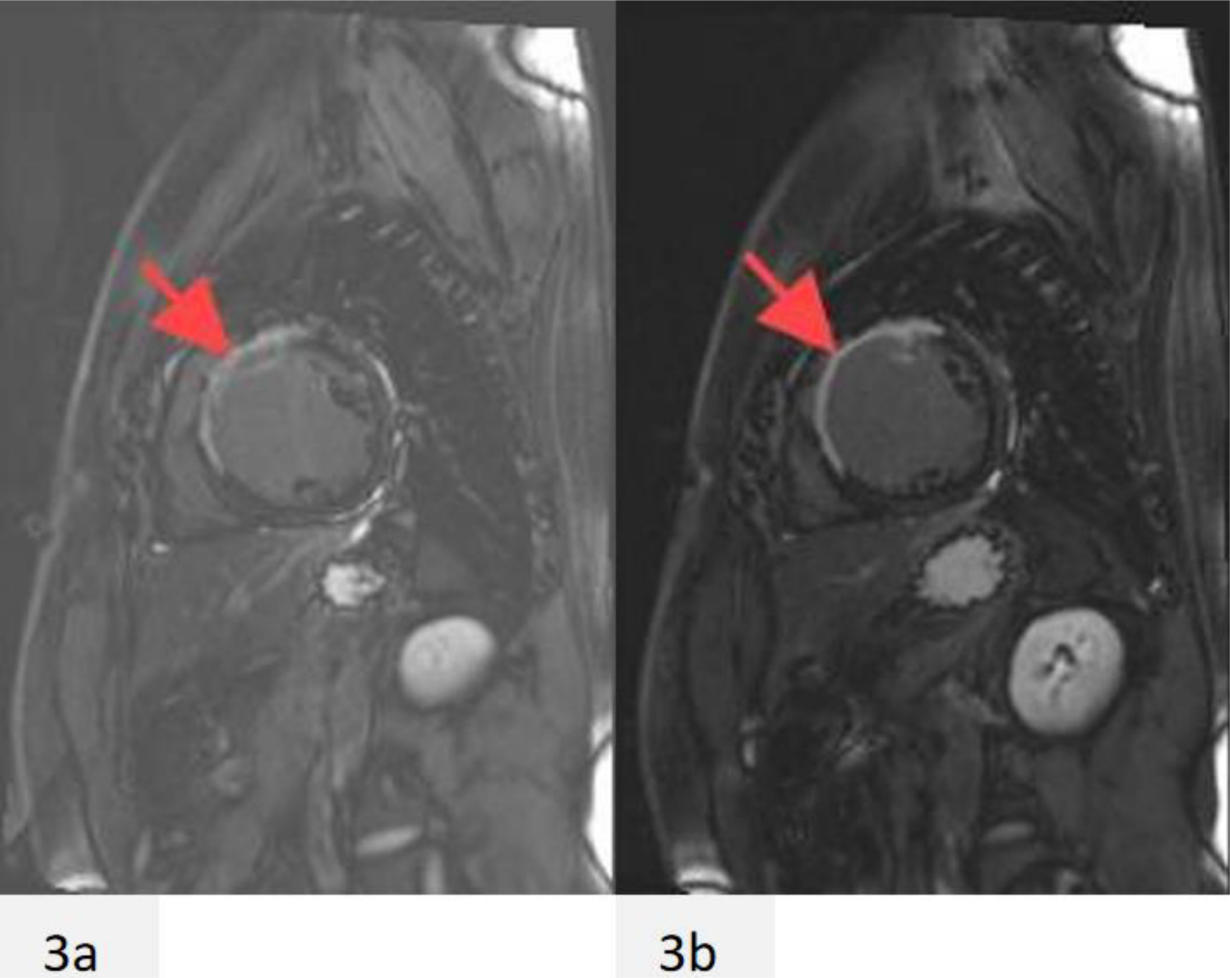
The patient underwent cardiac magnetic resonance imaging during their first admission **(a,b)**. The area indicated by the arrow in the figure represents cardiac scars or fibrosis.

There were no obvious abnormalities in the physical examination upon admission. Laboratory tests indicate a significant increase in myocardial injury markers (high-sensitivity troponin: 0.149 ng/ml, normal range: 0–0.014 ng/ml, myoglobin 141.8 ng/ml, normal range: 21–72 ng/ml, creatine kinase isoenzyme 23.14 ng/ml, normal range: 0–5.501 ng/ml). Blood cell count, serum electrolytes, liver and kidney function tests, and coagulation function are all within normal ranges. Electrocardiogram shows ST segment elevation in leads V2-V4, accompanied by ST segment depression in leads II, III, and aVF (Figure 1a), suggesting acute anterior wall ST segment elevation myocardial infarction. Subsequently, the patient underwent coronary angiography and found occlusion in the proximal segment of the left anterior descending artery (LAD) (Figure 1c), while no other coronary arteries were narrowed. Optical coherence tomography (OCT) examination results showed that the LAD lumen was mainly composed of organized thrombus, with the narrowest area of 0.98 mm^2^, an area stenosis rate of about 78.1%, and a diameter stenosis rate of about 53.3% (Figure 2a,b). One stent was implanted in series from far to near at the lesion site in the proximal and middle segments of the LAD (Figure 1d). At the same time, further improvements were made to lupus anticoagulants, autoantibody spectrum and ANCA, anti CCP antibodies, anti cardiolipin antibodies, and bilateral lower limb venous ultrasound, all of which were negative. We further investigated the possibility of thrombophilia. As expected, the patient carried two susceptibility genes, PAI-1 (4G/5G) and MTHFR (C > T) (Table 1). During hospitalization, the patient experienced frequent ventricular premature beats. A 24-hour dynamic electrocardiogram revealed 3,835 episodes of ventricular premature beats throughout the entire process. Cardiac magnetic resonance imaging showed weakened cardiac motion, enlarged left ventricular chamber, thinning of the middle and apical segments of the left ventricle, and a significant decrease in corresponding motion, mainly in the interventricular and anterior walls. Echocardiography shows a significant decrease in left ventricular systolic function (Table 1). The extensive thickening rate of the left ventricular wall decreased, mainly in the basal segment anterior interventricular and apical segment lateral walls (<0%). Left ventricular function: EF: 14.76% (Figure 3a,b). Subsequently, the patient received 100 mg of aspirin daily for antiplatelet aggregation therapy, 20 mg of atorvastatin for lipid-lowering therapy, and 20 mg of rivaroxaban for anticoagulant therapy.

**Table 1:** The patient carried two susceptibility genes, PAI-1 (4G/5G) and MTHFR (C > T).

Gene name	Genotype	Result	Reference allele	OR value
PAI-1 (4G/5G)	4G4G	−	5G	1.56
	4G5G	+		
	5G5G	−		
PROC (C > T)	CC	+	C	7.34
	CT	−		
	TT	−		
PROC (de1AAG)	DUP/DUP	+	DUP	2.71
	DEL/DUP	−		
	DEL/DEL	−		
THBD (G > T)	GG	+	G	2.8
	GT	−		
	TT	−		
MTHFR (C > T)	CC	−	C	1.75
	CT	+		
	TT	−		
APOH (T > C)	TT	+	T	1.55
	TC	−		
	CC	−		
SERPINC1 (G > A)	GG	+	G	1.2
	GA	−		
	AA	−		

During the 1 month follow-up after discharge, the patient still experienced recurrent palpitations, with approximately 3–4 episodes. When readmitted, high-sensitivity troponin levels were 0.038 ng/ml, normal range 0–0.014 ng/ml, myoglobin levels were 24.130 ng/ml, normal range 21–72 ng/ml, creatine kinase isoenzyme levels were 2.224 ng/ml, normal range 0–5.501 ng/ml, NT- proBNP levels were 1,976.00 pg/ml. NT- proBNP levels were significantly elevated compared to the previous admission (Table 2). The electrocardiogram showed paroxysmal ventricular tachycardia (Figure 1b), which was treated with amiodarone to control the arrhythmia. It was suggested that the patient undergo ICD implantation treatment, but the patient refused. After discharge, the patient was still treated with aspirin 100 mg, atorvastatin 20 mg, and rivaroxaban 20 mg.

**Table 2:** Two-dimensional data from the echocardiogram.

	First admission	Second admission
LA (mm)	38	34 × 52 × 43
LVDd (mm)	74	70
LVDs (mm)	58	58
IVS (mm)	9	8
LVPW (mm)	9	8
RA (mm)	50 × 41	48 × 45
RV (mm)	24	19
LVEF (%)	41	34

LA, left atrial diameter; LVDd, left ventricular end-diastolic diameter; LVDs, left ventricular end-diastolic diameter; IVS, interventricular septum; LVPW, left ventricular posterior wall at end-diastole; RA, suitable atrium diameter; RV, right ventricular diameter; LVEF, left ventricular ejection fraction.

## Discussion

It is recommended that young and middle-aged patients with severe coronary artery stenosis undergo screening for embolism. Currently, acquired or hereditary thrombophilia can be detected in many patients presenting with venous thromboembolism (VTE), and some patients may experience severe thromboembolic disease recurrence in the short term. The patient mainly presents with non induced lower limb venous thrombosis and short-term concurrent coronary thromboembolism, leading to acute myocardial infarction. The main causes of death in patients with acute myocardial death are malignant arrhythmia and significant decline in cardiac function due to excessive cardiac remodeling. We observed that the patient's myocardial infarction area was not large, but the heart function was extremely poor. Echocardiography shows a significant decline in cardiac function, while cardiac magnetic resonance imaging suggests severe cardiac scarring. Therefore, we speculate that thrombophilia may be an important cause of excessive cardiac remodeling leading to cardiac scars in young patients after myocardial infarction. The patient was prevented from further myocardial death due to myocardial ischemia after stent implantation treatment, but experienced irreversible serious complications. We suspect that hereditary thrombophilia may have some mechanism of action in vascular endothelial injury and endocardial damage.

The guidelines recommend indefinite antithrombotic therapy for most patients with non induced VTE. Research has shown that once anticoagulant therapy is discontinued, the risk of thromboembolic disease recurrence within 10 years after the first episode ranges from 30% to 50%. In this case report, the patient's self-discontinuation of oral anticoagulant therapy after the first occurrence of venous thrombosis may be one of the important reasons for coronary thrombosis.

Genetic thrombophilia is relatively rare in young patients with acute myocardial infarction caused by coronary artery thrombosis. Thrombophilia is not only an important pathogenesis leading to thromboembolism, but may also be a significant cause of adverse consequences after early onset of myocardial infarction. Therefore, in clinical practice, we need to be highly vigilant about the possibility of genetic thrombophilia in young patients with unexplained myocardial infarction.

## Conclusion

The changes in coagulation status caused by mutations in PAI-1 and MTHFR genes are the genetic basis of the patient's myocardial infarction. After stent implantation treatment, coronary blood flow was restored, and postoperative anticoagulation therapy was continued to prevent the formation of new blood clots. But the patient experienced severe arrhythmia and cardiac dysfunction, and the mechanism is currently unclear, which may be related to genetic thrombophilia.

## Data Availability

The original contributions presented in the study are included in the article/Supplementary Material, further inquiries can be directed to the corresponding author.
